# Biographic Clinics

**Published:** 1903-12

**Authors:** 


### Reviews of Books.

**Biographic Clinics.** By GEORGE M. GOULD, M.D. Pp. 223. London: Rebman, Ltd. 1903.

In his preface the author upbraids the medical profession for having confined its attention to but three aspects of disease, namely, healing the patient, learning from his case how to cure others, and investigating the cause of the disease irrespective of the individual patient. The study of the patient's "life-problem, . . . all of his diseases, past, present, *and to come*" (the italics are ours), which is scientifically more important, and which should be the splendid function of the family physician, is ignored. Dr. Gould contributes but little towards the attainment of this lofty aim. He plunges at once into a "medical description of past lives," choosing for his examples De Quincey, Carlyle, Darwin, Huxley and Browning. At first sight the choice seems to be an unfortunate one, since, as he admits, in each case "there is an almost utter lack of clinical data." But, as all readers of Dr. Gould's works will suspect, he is really in quest of only one *fons et origo mali*, astigmatism. The book is clever, interesting, and shows evidence of much labour and research, but the conclusions are illogical. We are assured that De Quincey had myopic astigmatism and exophoria which provoked depression of spirits, derangement of the liver, and a craving for opium. Carlyle, who attributed his wretchedness to "an infernal disorder of the stomach," was assured by Sir R. Quain that his complaint was due to the simultaneous indulgence in smoking and eating gingerbread—"very nasty gingerbread." We learn from Dr. Gould that astigmatism was the real cause, and that Darwin's almost historic liability to sea-sickness was due to refractive error. Huxley, who was convinced that his and the prophet Jeremiah's ailments were identical, suffered from flatulent dyspepsia due to compound hyperopic astigmatism, as also were Browning's "bilious headaches." The work concludes with an elaborate account of the discovery of astigmatism and the gradual recognition of its reflex effects. We cordially commend the book to the notice both of the general practitioner and the specialist, though probably neither will be convinced by the arguments put forth.

